# Resilience mediates the effect of the COVID-19 pandemic on mental health in a sample of adults in Panama

**DOI:** 10.3389/fpsyg.2023.1235935

**Published:** 2023-11-16

**Authors:** Diana C. Oviedo, Adam E. Tratner, María Sofía Pinzón, Sofía Rodríguez-Araña, Elianne Pauli-Quirós, Carlos Chavarría, Camilo Posada Rodríguez, Gabrielle B. Britton

**Affiliations:** ^1^Centro de Neurociencias y Unidad de Investigación Clínica, Instituto de Investigaciones Científicas y Servicios de Alta Tecnología (INDICASAT-AIP), Panama, Panama; ^2^Escuela de Psicología, Universidad Santa María la Antigua (USMA), Panama, Panama; ^3^Sistema Nacional de Investigación (SNI) SENACYT, Panama, Panama; ^4^Florida State University, Panama, Panama

**Keywords:** mental health, COVID-19, resilience, Latin America, mediation, stress, depression, anxiety

## Abstract

**Background:**

The COVID-19 pandemic was characterized by global increases in depression, anxiety, and stress symptoms. Previous studies have shown that resilience mitigates these symptoms, however there is limited research exploring the link between resilience and mental illness during the COVID-19 pandemic in Central America.

**Objective:**

To examine the role of resilience as it relates to the perceived effect of the pandemic on mental health symptoms.

**Methods:**

A sample of 480 adults in Panama were recruited from March to May 2021 to complete an online survey. The online survey consisted of sociodemographic questions and scale measures assessing depression, anxiety and stress symptoms, resilience, and social support.

**Results:**

Results indicated that resilience mediated the relationship between the perceived effect of the COVID-19 pandemic and mental health symptoms; participants who felt more personally affected by the pandemic reported more depression, anxiety, and stress symptoms via decreased resilience. Further analyses revealed that resilience was moderated by sex and social support, showing that the indirect effect of resilience was greater for women and individuals who perceived low social support.

**Discussion:**

These findings contribute to a growing body of research documenting the adverse effects of the COVID-19 pandemic on mental health and reveal potential mechanisms through which pandemic-related distress decreases resilience, thereby increasing symptoms of mental illness.

## 1. Introduction

The SARS-CoV-2 coronavirus disease 2019 (COVID-19) pandemic profoundly affected people around the world. At the height of the pandemic (i.e., January 2020 to December 2021) there were an estimated 14.83 million excess deaths worldwide (Msemburi et al., 2023). During this time period, most countries implemented strategies to reduce contagion, ranging from mask mandates and social distancing to strict lockdown measures such as quarantines, mobility restrictions, school suspensions, and border closures. Amidst health and safety concerns, the lockdowns disrupted social life and devastated many peoples’ livelihoods. These events coincided with global increases in depression, anxiety, stress, insomnia, and somatic symptoms (Huang et al., 2020; Majumdar et al., 2020; Olff et al., 2021; Mahmud et al., 2022). Hence, it has been argued that the pandemic precipitated a worldwide mental health crisis (Moreno et al., 2020; Tsamakis et al., 2021) that disproportionately affected certain vulnerable groups, such as young people (Cunningham et al., 2021), women, expectant mothers (Arzamani et al., 2022), people experiencing grief (Nohesara et al., 2022), people with preexisting mental health and chronic health conditions (Connor et al., 2020), people with limited access to social and health services (Clark et al., 2020), and people residing in low-and-middle income countries (Buitrago Ramírez et al., 2021; Ciria Villar and Día Sahún, 2021; González-Soto et al., 2021; Moya et al., 2021). Researchers have identified several protective factors that mitigated the adverse effects of the pandemic on mental health, such as social support, spiritual beliefs, self-efficacy, and a sense of purpose (Brailovskaia and Margraf, 2020; Cabanillas, 2020; Memaryan et al., 2021; Racine et al., 2022; Beck and Daniels, 2023; Diotaiuti et al., 2023). This article investigates the role of resilience in relation to the effect of the pandemic on mental health.

Resilience refers to the ability to adapt to adversity and recover from difficult experiences (Southwick et al., 2014). The construct of resilience comprises several interrelated psychological phenomena, which include stress tolerance, emotion regulation, cognitive appraisal, and self-efficacy (Herrman et al., 2011). That is, resilience stems from a combination of psychological and behavioral mechanisms that provide mental resources and strategies for navigating difficult experiences. Previous work has shown that high resilience buffers against acute stressors and facilitates post-traumatic growth (Carver, 1998; Connor and Davidson, 2003; Davidson et al., 2005; Wolmer et al., 2011), and it follows that individuals’ psychological response to the COVID-19 pandemic depended, in part, on resilience (Macías-Valadez Treviño et al., 2020). Indeed, research during the pandemic shows that higher resilience is associated with the use of healthy coping strategies, greater subjective well-being, and fewer symptoms of mental illness (Zhang et al., 2020; Finstad et al., 2021; Gundogan, 2021; Li et al., 2021; Verdolini et al., 2021).

Some scholars argue that social support was essential for cultivating resilience during the COVID-19 pandemic (PeConga et al., 2020). Social support involves the provisioning of assistance and comfort to individuals within relationship networks (e.g., friends, family, neighbors, etc.), and consists of behaviors such as physical aid, emotional support, advice, and companionship. There are several potential mechanisms through which social support increases resilience (Thoits, 2011). For instance, social support may promote resilience by creating safety networks, reducing loneliness and isolation, providing tangible resources (e.g., financial aid), and fostering optimism about the future. A large body of research has explored the connection between social support and psychological well-being (Uchino, 2006; Chu et al., 2010; Wang et al., 2018), including its role in mitigating psychological distress. Previous research has shown that people with higher self-perceived social support have less depression symptoms (Cohen and Wills, 1985; Cho et al., 2022) and lower risk of mortality (Turner and Lloyd, 1995) following a stressful life event, and have less severe trauma symptoms (Evans et al., 2013). Consequently, individuals who reported higher perceived social support during the COVID-19 pandemic had fewer depression, anxiety, and stress symptoms (Guo et al., 2021; Choi et al., 2022; Gabarrell-Pascuet et al., 2023). In sum, resilience may buffer against pandemic-related distress to the extent that individuals possess adequate social support.

Research during the pandemic has also documented sociodemographic differences in mental health that are attributed to differences in resilience. For instance, older adults reported less psychological distress than younger adults during the pandemic, in part because they are, on average, more resilient (Vahia et al., 2020; Mccleskey and Gruda, 2021). Because young adults experience loss and trauma with greater emotional intensity and have greater difficulty processing unpleasant emotions such as fear, anger, irritability and aggression (Ang et al., 2018; Viejo and Jesús, 2020; Martínez Arriaga et al., 2021), they may have been less resilient to the challenges presented by the pandemic. Individuals who experienced greater economic hardship during the pandemic reported more symptoms of mental illness and lower levels of resilience, perhaps due to increased uncertainty about the future or the inability to meet basic needs (Kimhi et al., 2020). Moreover, several studies suggest that women were more vulnerable to pandemic-related distress compared to men, resulting in worse mental health outcomes (Kumar et al., 2022; Manchia et al., 2022). Because women are more likely than men to develop stress-related psychological symptoms in response to traumatic events (North, 2016) and were disproportionately burdened with domestic and psychosocial responsibilities during the pandemic (e.g., childcare; Lowe et al., 2021), they may have experienced greater psychological distress that resulted in lower resilience (Hirani et al., 2016) and therefore more symptoms of mental illness.

Following previous research (Ye et al., 2020; Rossi et al., 2021; Ke et al., 2022; Noh and Park, 2022; Shi et al., 2022; Hirai et al., 2023; Park et al., 2023) the current study used a mediation approach to evaluate a potential mechanism through which pandemic-related stress decreases resilience, resulting in more symptoms of mental illness. In addition, this study explored the moderating effects of perceived social support and sex on resilience. The current study leveraged a sample of participants from a unique social context to examine the effect of the COVID-19 pandemic on mental health–Panama. Despite being one of the most affluent and developed countries in the Central American region, Panama had high rates of disease transmission and a high number of deaths per million inhabitants due to health complications from the virus (Pearson et al., 2021). Panama simultaneously implemented one of the strictest lockdowns in the world (Pescarini et al., 2020), which included curfews, severe mobility and travel restrictions, and suspension of most in-person activities, including compulsory education. These lockdown measures remained in effect until late 2021, with many restrictions continuing well into 2022. During the lockdown, Oviedo et al. (2022) documented a high prevalence of psychosocial disturbances, such as perceived isolation and strained social relationships, as well as poor mental health outcomes in a sample of Panamanian adults (Oviedo et al., 2022). Social support and resilience were both negatively associated with depression, anxiety, and stress symptoms, and women reported more depression, anxiety, and stress symptoms, suggesting that individuals’ response to pandemic-related stress depend on these factors in the Panamanian context.

### 1.1. Hypotheses

We hypothesized that resilience would mediate the relationship between the perceived effect of the COVID-19 pandemic and psychological symptoms. Specifically, individuals who feel more affected by the pandemic will report lower resilience, and more depression, anxiety, and stress symptoms through the indirect effect of resilience (*H1*). In addition, we hypothesized that perceived social support and sex would moderate the indirect effect of resilience. That is, individuals with lower perceived social support, particularly women, would also report lower resilience, and therefore more depression, anxiety, and stress symptoms through the indirect effect of resilience (*H2*).

## 2. Methods

### 2.1. Participants and procedure

Raosoft Sample Size Calculator was used to estimate an adequate sample size for the study. The estimated minimum sample size required was 323 participants given that there are 2,958,577 adults 18 and older in Panama (Contraloría General de la Nación, 2010), and the estimated prevalence rates of depression (50.9%), anxiety (57.4%), and stress (58.6%) during the pandemic (Bareeqa et al., 2021), with 95% confidence and 5% error. Convenience sampling was used to recruit a total of 480 adult residents in Panama (388 Women, 92 Men), aged 18 years or older (M = 32.7; SD = 14.6, Range = 18–66). A flyer was divulged on social media (e.g., Instagram, Twitter, Facebook, WhatsApp) to recruit participants. It contained a description of the study and contact information. Inclusion criteria included being 18 years or older, residing in Panama, having access to an electronic device such as a laptop, tablet, or cellphone, and not suffering from a physical condition that would make it difficult to access the survey or answer questionnaires (e.g., cognitive or visual impairment, illiteracy, etc.). Access to an online survey via a Google Forms link was sent to individuals who contacted a member of the research team. Participants were first prompted to participate by answering a few questions to verify they met the inclusion criteria. Data was collected from March to May 2021. This study was approved by the National Research Bioethics Committee of Panama (CNBI code PT-023). The participants signed informed consent in accordance with the Declaration of WMA (2023). The online survey included sociodemographic information such as age, sex, nationality, educational level, marital status, cohabitation, employment status, monthly household income, and scale measures of psychological constructs.

### 2.2. Measures

#### 2.2.1. Sociodemographic questionnaire

Participants reported their sex (0 = Female, 1 = Male), age, educational attainment (0 = Primary School, 1 = Secondary School, 2 = Technical Degree, 3 = Professional Licensure, 4 = Bachelor’s Degree, 5 = Post-Graduate Degree [Master’s or Doctorate]), monthly income in USD (0 = Less than $250, 1 = $250–$500, 2 = $500–$800, 3 = $800–$1,500, 4 = $1,500–$2,000, 5 = greater than $2000), and the number of cohabitants living with them in the same household (Table 1). Participants also indicated the extent to which they were personally affected by the COVID-19 pandemic (0 = Not at all affected, 1 = Affected very little, 2 = Affected, 3 = Greatly affected).

**Table 1:** Sociodemographic characteristics.

	Total (*n* = 480)	Female (*n* = 388)	Male (*n* = 92)
	*n* (%)/M (SD)	*n* (%)/M (SD)	*n* (%)/M (SD)
Sex			
Female	388 (80.8%)		
Male	92 (19.2%)		
Age	32.7 (14.6)	32.4 (14.3)	33.8 (15.6)
Nationality			
Panamanian	425 (88.5%)	344 (88.7%)	81 (88.0%)
Other	55 (11.5%)	44 (11.3%)	11 (12.0%)
Marital status			
Married or partnered	99 (20.6%)	77 (19.8%)	22 (23.9%)
Single, divorced, or widowed	381 (79.4%)	311 (80.2%)	70 (76.1%)
Educational attainment			
High school diploma	66 (13.8%)	48 (12.4%)	18 (19.6%)
Bachelor’s degree	235 (49.0%)	189 (48.7%)	46 (50.0%)
Graduate degree	130 (27.1%)	110 (28.4%)	20 (21.7%)
Employment status			
Unemployed	179 (37.3%)	144 (37.1%)	35 (38.0%)
Independent work	76 (15.8%)	63 (16.2%)	13 (14.1%)
Permanent contract	151 (31.5%)	121 (31.2%)	30 (32.6%)
Other	74 (15.4%)	60 (15.5%)	14 (15.2%)
Monthly household income			
$800–$1,500	94 (19.6%)	79 (20.4%)	15 (16.3%)
$1,500–$2,000	83 (17.3%)	65 (16.8%)	18 (19.6%)
>$2000	222 (46.3%)	177 (45.6%)	45 (48.9%)
Other	81 (16.8%)	67 (17.3%)	14 (15.2%)
Cohabitation			
Live alone	27 (5.6%)	21 (5.4%)	6 (6.5%)
2 Cohabitants	111 (23.1%)	89 (22.9%)	22 (23.9%)
3 Cohabitants	116 (24.2%)	93 (24.0%)	23 (25.0%)
4 Cohabitants	126 (26.3%)	103 (26.5%)	23 (25.0%)
5+ Cohabitants	100 (20.8%)	82 (21.1%)	18 (19.6%)

#### 2.2.2. Depression, anxiety, and stress scale-21

Participants reported how frequently they experienced depression, anxiety, and stress symptoms during the past week using the Depression, Anxiety and Stress Scale-21 (Bados et al., 2005). The DASS-21 consists of 21 Likert-scale items with response scores ranging from 0 to 3 (0 = It has not happened to me, 1 = It has happened to me a little or part of the time, 2 = It has happened to me quite a lot or for a good amount of the time, 3 = It has happened to me a lot, or most of the time) and contains three subscales with 7 items in each subscale. The depression subscale measures symptoms such as dysphoria, dulled senses, self-deprecation, loss of interest, and anhedonia. The anxiety subscale measures subjective and somatic symptoms of fear, autonomic activation, situational anxiety, and anxious attachment. The stress scale measures non-specific and persistent hypervigilance, difficulty relaxing, irritability, and impatience. Following previous research (Román et al., 2016), a sum score was calculated for each subscale, which produced scores ranging from 0 to 21. A total sum score of all 21 items was computed by aggregating the scores for all three subscales. Cronbach’s alpha indicated high internal consistency for the measure (α = 0.95).

#### 2.2.3. The Connor-Davidson resilience scale

Participants reported how psychologically resilient they felt during the past month using the Connor-Davidson Resilience Scale (Broche-Pérez et al., 2012). This scale consists of 25 Likert-scale items with responses ranging from 0 to 4 (0 = Never, 1 = Rarely, 2 = Sometimes, 3 = Often, 4 = Almost Always), and measures self-perceived competence, resolve, trust in one’s intuition, stress tolerance, positive acceptance to change, and locus of control. Examples of items include “I am able to adapt myself when changes arise” and “I remain focused and think clearly when under pressure.” A sum score was computed for all 25 items. Cronbach’s alpha indicated high internal consistency for the measure (α = 0.91).

#### 2.2.4. Multidimensional scale of perceived social support

Participants completed the Multidimensional Scale of Perceived Social Support (Ruiz-Jiménez et al., 2017), a 12-item measure that assesses the perceived quality of social support from family, friends, and relationship partners using a 5-point Likert scale (0 = Strongly disagree, 5 = Strongly agree). Examples of items include “My family really tries to help me” and “I can count on my friends when things aren’t going well.” A sum score was computed for all 12 items. Cronbach’s alpha indicated high internal consistency for the measure (α = 0.94).

A full list of items for each scale measure is provided in Supplemental Materials.

## 3. Results

### 3.1. Statistical analyses

**Figure 1:**
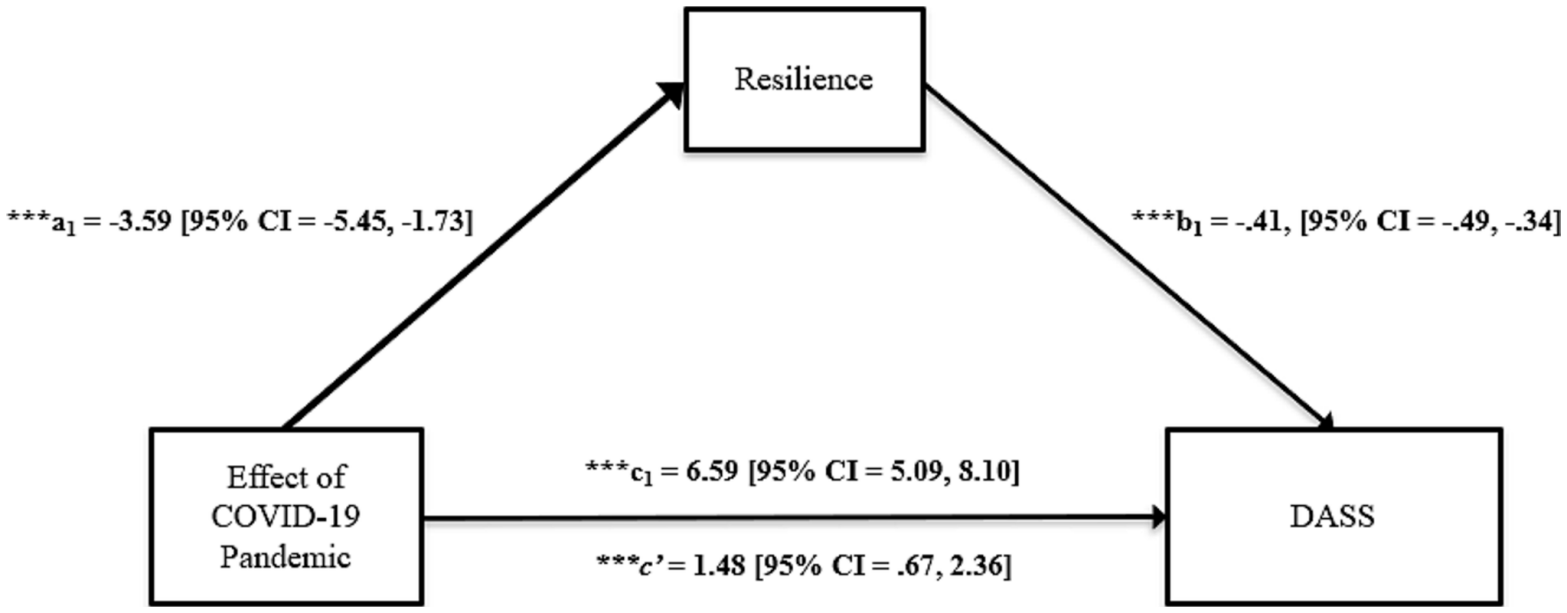
Simple mediation estimating the perceived effect of the COVID-19 pandemic on depression, anxiety, and stress symptoms through resilience. *p* < 0.05^*^, *p* < 0.01^**^, *p* < 0.001^***^.

**Figure 2:**
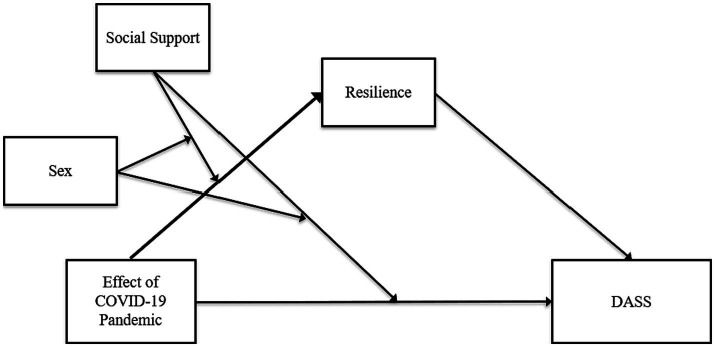
Conceptual illustration of the moderated mediation estimating the effect of the COVID-19 pandemic on depression, anxiety, and stress symptoms through resilience, moderated by social support and participant sex.

Five participants were removed from the dataset prior to analyses due to non-responses on scale measures. First, a correlation matrix was constructed using SPSS version 28 to inspect the associations between variables of interest and to report means and standard deviations. Next, a simple mediation analysis was conducted using the SPSS Macro PROCESS version 4.1 (Hayes, 2017). PROCESS model 4 was selected because it estimates the indirect path of the effect of the COVID-19 pandemic on depression, anxiety, and stress symptoms through resilience (see Figure 1). Lastly, a moderated-mediation analysis was conducted using PROCESS 4.1. PROCESS model 12 was selected for the moderated-mediation analysis because it estimates the indirect path of the effect of the COVID-19 pandemic on depression, anxiety, and stress symptoms through resilience, and simultaneously allows the effect of social support and sex to jointly moderate the indirect effect of resilience. A conceptual illustration of the moderated mediation model is shown in Figure 2.

For each mediation model, the mean score of the effect of the COVID-19 pandemic was entered as the focal antecedent variable (i.e., *X*), the sum score of resilience was entered as the mediator variable (i.e., *M*), and the sum score of depression, anxiety, and stress symptoms (DASS) was entered as the outcome variable (i.e., *Y*). Sex, age, educational attainment, monthly income, and the number of cohabitants in the same household were entered as covariates in the simple mediation model to adjust for their effects on depression, anxiety, and stress symptoms. The moderated-mediation model included the sum score of social support as a moderator (i.e., W) and sex (male, female) as a dichotomous moderator (i.e., *Z*), and age, educational attainment, monthly income, and the number of cohabitants in the same household as covariates. Two-way interaction terms were computed for the effect of the COVID-19 pandemic and social support (*X × W*), the effect of the COVID-19 pandemic and sex (*X × Z*), social support and sex (*W × Z*), as well as a three-way interaction term using the effect of the COVID-19 pandemic, social support, and sex (*X × W* × *Z*). Mediation was inferred from the indirect path via the predictors (e.g., effect of the pandemic, sex, social support) to the outcome (DASS) through the mediator (resilience), and significance was determined based on boot-strapped confidence intervals of the indirect effects. Moderation of mediation was determined if the slopes of the moderators (sex, social support) defining the size of the indirect effects were different from zero (i.e., the indices of moderated mediation) using bootstrapped confidence intervals (Hayes, 2015).

### 3.2. Zero-order correlations

A correlation matrix was constructed to investigate zero-order relationships between sociodemographic variables, depression, anxiety, and stress symptoms (DASS), resilience, social support, and the effect of the COVID-19 pandemic (Table 1). Results and descriptive statistics are summarized in Table 2.

**Table 2:** Correlations between sociodemographic variables and depression, anxiety, and stress symptoms, resilience, social support, and the effect of the COVID-19 pandemic.

Variable	1	2	3	4	5	6	7	8	9
1. Sex	___								
2. Age	0.04	___							
3. Education	−0.06	0.33^***^	___						
4. Income	0.04	0.10^*^	0.25^***^	___					
5. Effect of Pandemic	−0.02	−0.10^*^	−0.03	−0.15^***^	___				
6. DASS	−0.14^**^	−0.33^***^	−0.12^**^	0.09^*^	0.42^***^	___			
7. Resilience	0.09	0.35^***^	0.18^***^	0.15^**^	−0.21^***^	−0.54^***^	___		
8. Social Support	−0.04	0.05	0.05	0.15^**^	−0.10^*^	−0.26^***^	0.36^***^	___	
9. Cohabitation	−0.02	−0.25^***^	−0.21^***^	−0.15^***^	0.16^***^	0.16^***^	−0.14^**^	0.012	___
Mean	N/A	32.78	3.80	3.82	1.72	19.22	71.59	67.05	2.54
Standard Deviation	N/A	14.65	1.13	1.40	0.66	13.74	14.21	15.33	1.52

*p* < 0.05^*^, *p* < 0.01^**^, *p* < 0.001^***^; Zero-order correlations and descriptive statistics for participant sex, age, educational attainment, monthly income, depression, anxiety, and stress symptoms (DASS), resilience, social support, and the effect of the COVID-19 pandemic.

### 3.3. Simple mediation analysis

Estimated regression coefficients and statistical models of direct and indirect effects are presented in Tables 3 and 4 and illustrated in Figure 1. Results indicated that the effect of the COVID-19 pandemic on DASS score was negatively associated with resilience, such that participants who were more affected by the pandemic scored lower on resilience relative to those who were less affected by the pandemic (a_1_ = −3.59 [95% CI = −5.45, −1.73], *p* < 0.001). Holding constant the effect of the pandemic, resilience was negatively associated with DASS, such that participants who reported greater resilience reported lower DASS relative to those who reported lower resilience (b_1_ = −0.41, [95% CI = −0.49, −0.34], *p* < 0.001). There was a direct effect of the pandemic on DASS (c_1_ = 6.59 [95% CI = 5.09, 8.10], *p* < 0.001), indicating that participants who were more affected by the pandemic experienced greater depression, anxiety, and stress symptoms. Furthermore, the indirect effect of resilience mediated the effect of the pandemic on DASS (*c’* = 1.48 [95% CI = 0.67, 2.36]). These findings supported the first hypothesis, namely, that participants who felt more affected by the COVID-19 pandemic reported lower resilience, which predicted greater depression, anxiety, and stress symptoms.

**Table 3:** Simple mediation estimating the perceived effect of the COVID-19 pandemic on depression, anxiety, and stress symptoms through resilience.

	Resilience (*M*) coefficient	95% CI	DASS (*Y*) coefficient	95% CI
Effect of Pandemic (*X*)	a_1_–3.59 (0.95)^***^	−5.45, −1.73	c_1_ 6.59 (0.77)^***^	5.09, 8.10
Resilience (*M*)			b_1_–0.41 (0.04)^***^	−0.49, −0.34
Sex	2.28 (1.56)	−0.78, 5.35	−3.38 (1.25)^**^	−5.84, −0.93
Age	0.29 (0.04)^***^	0.20, 0.38	−0.13 (0.04)^***^	−0.21, −0.06
Education	1.85 (1.24)	−0.59, 4.29	−0.67 (0.10)	−2.63, 1.28
Income	0.75 (0.47)	−0.18, 1.68	0.46 (0.38)	−0.29, 1.20
Cohabitation	0.01 (0.43)	−0.84, 0.85	0.12 (0.34)	−0.56, 0.79
Constant	58.86 (4.75)^***^	49.53, 68.19	42.65 (4.40)^***^	34.01, 51.30
	*R^2^* = 0.17		*R^2^* = 0.43	
	*F* (6, 445) = 14.86^***^		*F* (7, 444) = 48.26^***^	

*p* < 0.01^**^, *p* < 0.001^***^; standard errors in parentheses. Unstandardized regression coefficients, standard errors, and confidence intervals estimating the effect of the COVID-19 pandemic on depression, anxiety, and stress symptoms (DASS) through resilience, controlling for sex, age, educational attainment, monthly income, and number of cohabitants.

**Table 4:** Simple Mediation: Total, direct, and indirect effects of the perceived effect of the COVID-19 pandemic on depression, anxiety, and stress symptoms through resilience.

Total effect	Direct effect	Indirect effect
Coefficient *t*-value	Coefficient *t*-value	Effect 95% CI
8.07 (0.85)^***^ 9.50	6.59 (0.77)^***^ 8.59	1.48 (0.43)^***^ 0.67, 2.36

Boot-strapped confidence interval does not include zero^***^.

### 3.4. Moderated-mediation analysis

Estimated regression coefficients and statistical models are presented in Table 5. Results indicated that there was an effect of the COVID-19 pandemic (a_1_ = −0.62 [95% CI = −4.60, −0.65], *p* < 0.01) and social support (a_2_ = 0.33, [95% CI = 0.25, 0.42], *p* < 0.001), and a two-way interaction of the effect of the pandemic and social support on resilience (a_4_ = 0.18, [95% CI = 0.05, 0.30], *p* < 0.01), such that participants who were more affected by the pandemic and who perceived less social support reported lower resilience. After including sex and social support in the model, the moderated mediation analysis only explained an additional ~2% of the variance in depression, anxiety, and stress symptoms compared to the simple mediation analysis. However, the inclusion of social support explained an additional ~13% of the variance in resilience and revealed both direct and indirect effects on depression, anxiety, and stress symptoms.

**Table 5:** Moderated mediation estimating the effect of the COVID-19 pandemic on depression, anxiety, and stress symptoms through resilience, moderated by social support and participant sex.

	Resilience (*M*) coefficient	95% CI	DASS (*Y*) coefficient	95% CI
Effect of Pandemic (*X*)	a_1_–2.62 (1.01)^**^	−4.60, −0.65	c_1_ 7.41 (0.87)^***^	5.70, 9.12
Social Support (*W*)	a_2_ 0.33 (0.04)^***^	0.25, 0.42	c_2_ -0.08 (0.04)^*^	−0.16, −0.01
Sex (*Z*)	a_3_ 2.55 (1.44)	−0.29, 5.38	c_3_ -3.59 (1.24)^**^	−6.04, −1.15
*X* × *W*	a_4_ 0.18 (0.07)^**^	0.05, 0.30	c_4_ -0.12 (0.06)^*^	−0.23, −0.01
*X* × *Z*	a_5_ 0.82 (2.07)	−3.25, 4.89	c_5_ -3.83 (1.78)^*^	−7.33, −0.34
*W* × *Z*	a_6_–0.12 (0.09)	−0.29, 0.06	c_6_ 0.02 (0.08)	−0.13, 0.17
*X* × *W* × *Z*	a_7_ 0.13 (0.11)	−0.09, 0.34	c_7_ 0.05 (0.09)	−0.13, 0.24
Resilience (*M*)			b_1_–0.37 (0.04)^***^	−0.45, −0.28
Age	0.30 (0.04)^***^	0.22, 0.38	−0.15 (0.04)^***^	−0.22, −0.07
Education	1.13 (1.15)	−1.13, 3.39	−0.45 (0.99)	−2.40, 1.49
Income	0.23 (0.44)	−0.63, 1.10	0.57 (0.38)	−0.17, 1.32
Cohabitation	−0.09 (0.40)	−0.87, 0.70	0.15 (0.34)	−0.52, 0.82
Constant	57.23 (4.09)^***^	49.20, 65.26	49.76 (4.22)^***^	41.47, 58.06
	*R^2^* = 0.30		*R^2^* = 0.45	
	*F* (11, 440) = 17.40^***^		*F* (12, 439) = 29.96^***^	

*p <* 0.05^*^, *p* < 0.01^**^, *p* < 0.001^***^; Standard errors in parentheses. Unstandardized regression coefficients, standard errors, and confidence intervals estimating the effect of the COVID-19 pandemic on depression, anxiety, and stress symptoms (DASS) through resilience, moderated by social support and participant sex. Age, educational attainment, monthly income, and number of cohabitants were entered as covariates.

**Figure 3:**
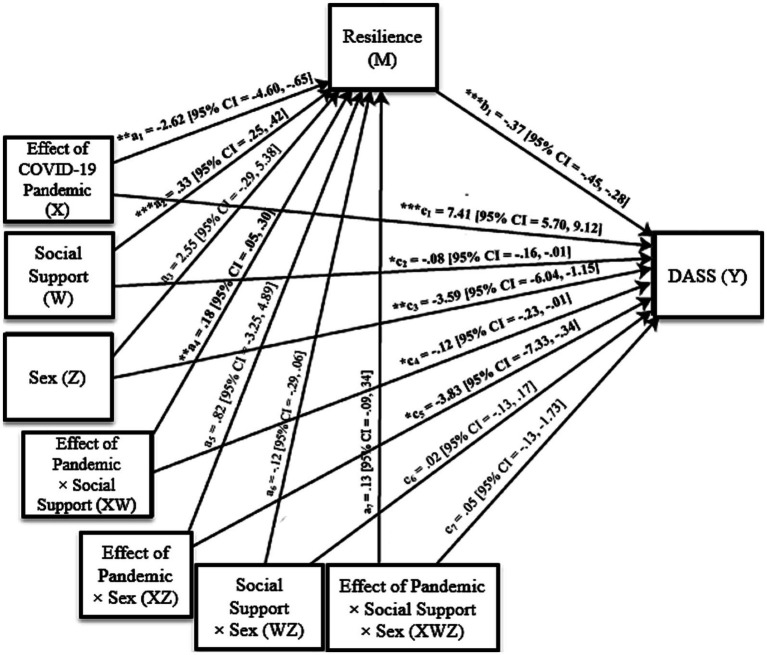
Moderated mediation estimating the direct effect of the COVID-19 pandemic on depression, anxiety, and stress symptoms through resilience, moderated by social support and participant sex. *p* < 0.05^*^, *p* < 0.01^**^, *p* < 0.001^***^.

The effect of sex, the two-way interaction between the effect of the pandemic and sex, the two-way interaction between sex and social support, and the three-way interaction between the effect of the pandemic, sex, and social support were not significantly related to resilience. Holding constant the effect of the pandemic, sex, social support, and all 2-way and 3-way interactions, resilience was negatively associated with DASS, such that participants who scored higher in resilience reported less depression, anxiety, and stress symptoms (b_1_ = −0.37, [95% CI = −0.45, −0.28], *p* < 0.001). There was a direct effect of the pandemic (c_1_ = 7.41, [95% CI = 5.70, 9.12], *p* < 0.001), social support (c_2_ = −0.08, [95% CI = −0.16, −0.01], *p* < 0.05), sex (c_3_ = −3.59, [95% CI = −6.04, −1.15], *p* < 0.01), a two-way interaction of the effect of the pandemic and social support (c_4_ = −0.12, [95% CI = −0.23, −0.01], *p* < 0.05), and a two-way interaction of the effect of the pandemic and sex (c_5_ = −3.83, [95% CI = −7.33, −0.34], *p* < 0.05) on DASS. The two-way interaction between sex and social support, and the three-way interaction between the effect of the pandemic, sex, and social support were not significantly related to DASS. Conditional direct effects split by sex and social support are summarized in Table 6 and illustrated in Figure 3.

**Table 6:** Moderated Mediation: Conditional direct effects of the COVID-19 pandemic on depression, anxiety, and stress symptoms, split by sex and social support.

Sex	Social support	Effect	Standard error	*t*	95% CI
Female	Low	9.00^***^	1.14	7.92	6.77, 11.23
Male	Low	4.46^**^	1.56	2.85	1.38, 7.53
Female	Moderate	6.98^***^	0.89	7.81	5.22, 8.74
Male	Moderate	3.34^*^	1.66	2.01	0.07, 6.61
Female	High	5.70^***^	1.18	4.82	3.38, 8.02
Male	High	2.62	2.14	1.23	−1.57, 6.82

*p* < 0.05^*^, *p* < 0.01^**^, *p* < 0.001^***^.

The moderated mediation analysis also yielded indirect effects of the pandemic on DASS through resilience. Although the index of moderated-moderated mediation was not significant [95% CI = −0.14, 0.06], the indices of conditional moderated mediation were significant for both men [95% CI = −0.19, −0.02] and women [95% CI = −0.13, −0.003], indicating that the mediator (resilience) varied as a function of perceived social support for both sexes. Specifically, women with low (*b* = 1.79, [95% CI = 0.50, 3.23]) and moderate (*b* = 0.73, [95% CI = 0.03, 1.51]) perceived social support, and men with low perceived social support (*b* = 2.09, [95% CI = 0.53, 3.70]) scored higher in DASS via lower resilience (see Tables 7–9). These findings support the second hypothesis by showing that participants who felt more affected by the COVID-19 pandemic and perceived less social support reported lower resilience, which predicted greater depression, anxiety, and stress symptoms. Further, the results suggest that the moderating effect of social support on resilience was more pronounced for women.

**Table 7:** Moderated mediation: conditional indirect effects of the COVID-19 pandemic on depression, anxiety, and stress symptoms, split by sex and social support.

Sex	Social support	Effect	Standard error	95% CI
Female	Low	1.79^***^	0.68	0.50, 3.23
Male	Low	2.09^***^	0.79	0.53, 3.70
Female	Moderate	0.73^***^	0.37	0.03, 1.51
Male	Moderate	0.27	0.67	−1.00, 1.66
Female	High	0.06	0.46	−0.89, 1.01
Male	High	−0.88	0.90	−2.52, 1.03

Boot-strapped confidence interval does not include zero^***^.

**Table 8:** Index of moderated-moderated mediation: social support and sex as joint moderators of resilience.

Index	Standard error	95% CI
−0.05	0.05	−0.14, 0.06

**Table 9:** Indices of conditional moderated mediation: social support as a moderator of resilience, split by sex.

Sex	Index	Standard error	95% CI
Female	−0.06^***^	0.03	−0.13, −0.003
Male	−0.11^***^	0.04	−0.19, −0.02

Boot-strapped confidence interval does not include zero^***^.

## 4. General discussion

This study examined the relationships between COVID-19 pandemic-related stress, resilience and social support, and mental health in a sample of adults in Panama. The goal of this research was to examine the role of resilience as it relates to the perceived effect of the pandemic on mental health symptoms, and whether resilience depends on perceived social support. Preliminary correlation analyses identified demographic and psychosocial factors associated with mental health. In line with previous research (Kimhi et al., 2020), results showed that older, more educated, and more affluent adults reported higher resilience. Being female, poorer, less educated, and having low resilience and social support was associated with increased depression, anxiety, and stress symptoms.

The main analysis investigated whether resilience mediates the association between the perceived effect of the pandemic and self-reported mental health symptoms, revealing both direct and indirect pathways from pandemic-related stress to depression, anxiety, and stress symptoms. Holding constant several demographic variables, individuals who felt more affected by the pandemic reported more depression, anxiety, and stress symptoms, and resilience mediated the association between the effect of the pandemic and mental health symptoms. Inversely, individuals with higher levels of resilience showed less of these symptoms. These results provided support for the first hypothesis. A moderated-mediation analysis similarly showed that perceived effect of the pandemic on mental health symptoms was mediated by resilience, but also indicated that the indirect effect of resilience was moderated by social support and sex, which supported the second hypothesis. Specifically, the indirect effect of resilience was greater for men with lower perceived social support, and women with low and moderate perceived social support. The mediation analyses also yielded direct effects showing that individuals who felt more affected by the pandemic and less resilient reported more depression, anxiety, and stress symptoms. Furthermore, the direct effect of the pandemic on mental health symptoms was greater for women and for individuals who perceived less social support.

In line with previous studies, these findings suggest that resilience may play an important role in coping with pandemic-related distress (Wolmer et al., 2011; Ke et al., 2022). That is, individuals’ psychological adjustment to the pandemic depended, in part, on the perception that they were capable of coping with the pandemic (i.e., resilience). It is possible that highly resilient individuals viewed the pandemic as less impactful or felt more capable of overcoming its challenges, which resulted in less distress and fewer symptoms of mental illness. Alternatively, people who felt more affected by the pandemic may have become less resilient to the ongoing stressors, thereby resulting in poorer mental health. Further, the results show that low psychological resilience, low social support, and sex are significant risk factors for negative mental health outcomes during the pandemic.

This study offers several contributions to the literature on the psychosocial effects of the COVID-19 pandemic. For instance, the current study replicated existing research on the mediating effect of resilience on psychological distress and mental health symptoms, (Bareeqa et al., 2021) the negative association between perceived social support and mental health symptoms (Bados et al., 2005; Román et al., 2016), and sex differences in mental health symptoms (Broche-Pérez et al., 2012; Hayes, 2015; Hayes, 2017; Ruiz-Jiménez et al., 2017). On one hand, the results show that low psychological resilience and low social support are significant risk factors for negative mental health outcomes. On the other hand, these findings suggest that social support is an underlying component of psychological resilience as it relates to COVID-19 pandemic distress and mental health symptoms.

### 4.1. Implications

This research has implications for theory and practice. First, the study supports the predictions of Resilience Theory (Greene et al., 2004), which proposes that different promotive psychosocial factors, such as social support can mitigate the psychological distress associated with traumatic experiences and is an essential component of resilience. Our analyses showed that perceived social support explained significant variation in resilience, which supports the idea that resilience is enhanced by supportive interpersonal relationships and can help individuals maintain well-being amidst difficult circumstances (Southwick et al., 2014). Second, the results corroborate the prediction that the pandemic disproportionately impacted certain vulnerable groups, such as people with lower socioeconomic status, less education, younger adults, and women. Third, this study can help mental health professionals in developing psychoeducational resources that reduce the psychosocial impact of COVID-19 by implementing healthy coping behaviors and cultivating resilience (Diotaiuti et al., 2021; D’Oliveira et al., 2022; da Cruz et al., 2022). One potential path forward from the COVID-19 pandemic is to implement mental health interventions that emphasize social support — particularly for women — to mitigate the harmful effects of pandemic-related stress. Finally, this study contributes to the literature on the psychosocial effects of the COVID-19 pandemic by replicated existing research on the mediating effect of resilience on psychological distress and mental health symptoms (Tuxunjiang et al., 2022), the negative association between perceived social support and mental health symptoms (Grey et al., 2020; Caccia et al., 2021), and sex differences in mental health symptoms (Dubey et al., 2020; Wang et al., 2020; Callís-Fernández et al., 2021; Jawad et al., 2021).

### 4.2. Limitations

The design of this study was correlational and cross-sectional, which precludes any causal inferences about how resilience underlies pandemic-related stress and mental health symptoms. We contend that the mediation analyses were appropriate for these data given that the construct of resilience is a relatively stable trait (Herrman et al., 2011), whereas mental health symptoms can fluctuate in response to stressors (e.g., the COVID-19 lockdown). Additionally, participants were recruited via convenience sampling, and therefore were not representative of the Panamanian population. However, restrictions at the time of data collection did not permit in-person recruitment or direct contact with local communities. Despite these limitations, these data contribute to a growing body of research documenting the adverse effects of the COVID-19 pandemic on mental health in Latin America and is among the first studies conducted in Central America on this topic.

## 5. Conclusion

The current research explored the role of resilience as it relates to the impact of the COVID-19 pandemic on mental health. Resilience plays an important role in individuals’ psychosocial response to the pandemic, and social support may be essential for cultivating resilience during the pandemic. This study corroborates recent research documenting that high resilience coupled with strong social support is associated with better mental health outcomes. This study also corroborates other recent studies documenting that resilience and perceived social support are uniquely associated with depression, anxiety, and stress symptoms, independent of socioeconomic and other demographic variables. In conclusion, the study contributes to a growing body of research documenting the adverse effects of the COVID-19 pandemic on mental health in Latin America and is among the first studies conducted in Central America on this topic.

## Data availability statement

The raw data supporting the conclusions of this article will be made available by the authors, without undue reservation.

## Ethics statement

The studies involving humans were approved by National Research Bioethics Committee of Panama (CNBI code PT-023). The studies were conducted in accordance with the local legislation and institutional requirements. The participants provided their written informed consent to participate in this study.

## Author contributions

DCO and AET: conceived and wrote the manuscript. MSP, SR-A, EP-Q, CC, CPR and GBB: read, reviewed, wrote sections, and equally contributed to the intellectual content and format of the manuscript. All authors contributed to the article and approved the submitted version.
