# Supplementary material for: Resilience mediates the effect of the COVID-19 pandemic on mental health in a sample of adults in Panama
Source: Front Psychol. 2023 Nov 16;14:1235935. doi: 10.3389/fpsyg.2023.1235935 (PMC10687484; doi:10.3389/fpsyg.2023.1235935)
Supplement: Supplementary file 1 [file Data_Sheet_1.docx]

## **SUPPLEMENTAL MATERIALS**

## **ESCALA DE DEPRESIÓN ANSIEDAD Y ESTRÉS -21 (DASS-21)**

Por favor lea las siguientes afirmaciones y marque el número (0, 1, 2, 3) que indica en qué grado le ha ocurrido a usted esta afirmación **durante la semana pasada.** La escala de calificación es la siguiente:

- **0: No me ha ocurrido.**
- **1: Me ha ocurrido un poco, o durante parte del tiempo.**
- **2: Me ha ocurrido bastante, o durante una buena parte del tiempo.**
- **3: Me ha ocurrido mucho, o la mayor parte del tiempo.**

| Afirmaciones | 0 | 1 | 2 | 3 |
| --- | --- | --- | --- | --- |
| 1. Me ha costado mucho descargar la tensión. |  |  |  |  |
| 1. Me di cuenta que tenía la boca seca. |  |  |  |  |
| 1. No podía sentir ningún sentimiento positivo. |  |  |  |  |
| 1. Se me hizo difícil respirar. |  |  |  |  |
| 1. Se me hizo difícil tomar la iniciativa para hacer cosas. |  |  |  |  |
| 1. Reaccioné exageradamente en ciertas situaciones. |  |  |  |  |
| 1. Sentí que mis manos temblaban. |  |  |  |  |
| 1. He sentido que estaba gastando una gran cantidad de energía. |  |  |  |  |
| 1. Estaba preocupado por situaciones en las cuales podía tener pánico o en las que podría hacer el ridículo. |  |  |  |  |
| 1. He sentido que no había nada que me ilusionara. |  |  |  |  |
| 1. Me he sentido inquieto. |  |  |  |  |
| 1. Se me hizo difícil relajarme. |  |  |  |  |
| 1. Me sentí triste y deprimido. |  |  |  |  |
| 1. No toleré nada que no me permitiera continuar con lo que estaba haciendo. |  |  |  |  |
| 1. Sentí que estaba al punto de pánico. |  |  |  |  |
| 1. No me pude entusiasmar por nada. |  |  |  |  |
| 1. Sentí que valía muy poco como persona. |  |  |  |  |
| 1. He tendido a sentirme enfadado con facilidad. |  |  |  |  |
| 1. Sentí los latidos de mi corazón a pesar de no haber hecho ningún esfuerzo físico. |  |  |  |  |
| 1. Tuve miedo sin razón. |  |  |  |  |
| 1. Sentí que la vida no tenía ningún sentido. |  |  |  |  |

**DEPRESSION, ANXIETY AND STRESS SCALE - 21 ITEMS (DASS-21)**

Please read each statement and circle a number 0, 1, 2 or 3 which indicates how much the statement applied to you over the past week. There are no right or wrong answers.

The rating scale is as follows:

**0 Did not apply to me at all**

**1 Applied to me to some degree, or some of the time**

**2 Applied to me to a considerable degree or a good part of time**

**3 Applied to me very much or most of the time.**

| \| Affirmations \| 0 \| 1 \| 2 \| 3 \| \| --- \| --- \| --- \| --- \| --- \| \| 1. I found it hard to wind down. \|  \|  \|  \|  \| \| 1. I was aware of dryness of my mouth. \|  \|  \|  \|  \| \| 1. I couldn’t seem to experience any positive feeling at all \|  \|  \|  \|  \| \| 1. I experienced breathing difficulty (e.g. excessively rapid breathing, breathlessness in the absence of physical exertion). \|  \|  \|  \|  \| \| 1. I found it difficult to work up the initiative to do things. \|  \|  \|  \|  \| \| 1. I tended to over-react to situations. \|  \|  \|  \|  \| \| 1. I experienced trembling (e.g. in the hands). \|  \|  \|  \|  \| \| 1. I felt that I was using a lot of nervous energy. \|  \|  \|  \|  \| \| 1. I was worried about situations in which I might panic and make a fool of myself. \|  \|  \|  \|  \| \| 1. I felt that I had nothing to look forward to. \|  \|  \|  \|  \| \| 1. I found myself getting agitated. \|  \|  \|  \|  \| \| 1. I found it difficult to relax. \|  \|  \|  \|  \| \| 1. I felt down-hearted and blue. \|  \|  \|  \|  \| \| 1. I was intolerant of anything that kept me from getting on with what I was doing. \|  \|  \|  \|  \| \| 1. I felt I was close to panic. \|  \|  \|  \|  \| \| 1. I was unable to become enthusiastic about anything. \|  \|  \|  \|  \| \| 1. I felt I wasn’t worth much as a person. \|  \|  \|  \|  \| \| 1. I felt that I was rather touchy. \|  \|  \|  \|  \| \| 1. I was aware of the action of my heart in the absence of physical exertion (e.g. sense of heart rate increase, heart missing a beat). \|  \|  \|  \|  \| \| 1. I felt scared without any good reason. \|  \|  \|  \|  \| \| 1. I felt that life was meaningless. \|  \|  \|  \|  \| |
| --- | --- | --- | --- | --- | --- | --- | --- | --- | --- | --- | --- | --- | --- | --- | --- | --- | --- | --- | --- | --- | --- | --- | --- | --- | --- | --- | --- | --- | --- | --- | --- | --- | --- | --- | --- | --- | --- | --- | --- | --- | --- | --- | --- | --- | --- | --- | --- | --- | --- | --- | --- | --- | --- | --- | --- | --- | --- | --- | --- | --- | --- | --- | --- | --- | --- | --- | --- | --- | --- | --- | --- | --- | --- | --- | --- | --- | --- | --- | --- | --- | --- | --- | --- | --- | --- | --- | --- | --- | --- | --- | --- | --- | --- | --- | --- | --- | --- | --- | --- | --- | --- | --- | --- | --- | --- | --- | --- | --- | --- | --- |

**ESCALA DE RESILIENCIA DE CONNOR-DAVIDSON (CD-RISC)**

Indique cuál es su grado de acuerdo con las siguientes afirmaciones o caso durante el último mes. Si alguna en particular no le ha ocurrido, responda según crea que se hubiera sentido. Utilice para ello la siguiente escala:

- **0: Nunca.**
- **1: Rara vez.**
- **2: A veces.**
- **3: A menudo.**
- **4: Casi siempre.**

| Afirmaciones | 0 | 1 | 2 | 3 | 4 |
| --- | --- | --- | --- | --- | --- |
| 1. Soy capaz de adaptarme cuando surgen cambios. |  |  |  |  |  |
| 1. Tengo al menos una relación íntima y segura que me ayuda cuando estoy estresado (a). |  |  |  |  |  |
| 1. Cuando no hay soluciones claras a mis problemas, a veces la suerte o Dios puede ayudarme. |  |  |  |  |  |
| 1. Puedo enfrentarme a cualquier cosa. |  |  |  |  |  |
| 1. Los éxitos del pasado me dan confianza para enfrentarme a nuevos desafíos y dificultades. |  |  |  |  |  |
| 1. Cuando me enfrento a los problemas intento ver su lado gracioso. |  |  |  |  |  |
| 1. Enfrentarme a las dificultades puede hacerme más fuerte. |  |  |  |  |  |
| 1. Tengo tendencia a recuperarme pronto luego de enfermedades, heridas u otras dificultades. |  |  |  |  |  |
| 1. Buenas o malas, creo que la mayoría de las cosas ocurren por alguna razón. |  |  |  |  |  |
| 1. Siempre me esfuerzo sin importar cuál pueda ser el resultado. |  |  |  |  |  |
| 1. Creo que puedo lograr mis objetivos, incluso si hay obstáculos. |  |  |  |  |  |
| 1. No me doy por vencido (a), aunque las cosas parezcan no tener solución. |  |  |  |  |  |
| 1. Durante los momentos de estrés o crisis, sé dónde puedo buscar ayuda. |  |  |  |  |  |
| 1. Bajo presión, me mantengo enfocado/a y pienso claramente. |  |  |  |  |  |
| 1. Prefiero intentar solucionar las cosas por mí mismo, a dejar que otros decidan por mí. |  |  |  |  |  |
| 1. No me desanimo fácilmente ante el fracaso. |  |  |  |  |  |
| 1. Creo que soy una persona fuerte cuando me enfrento a los desafíos y dificultades vitales. |  |  |  |  |  |
| 1. Si es necesario, puedo tomar decisiones difíciles que podrían afectar a otras personas. |  |  |  |  |  |
| 1. Soy capaz de manejar sentimientos desagradables o dolorosos: Ej. tristeza, temor y enfado. |  |  |  |  |  |
| 1. Al enfrentarse a los problemas a veces hay que actuar intuitivamente (sin saber por qué). |  |  |  |  |  |
| 1. Tengo un fuerte sentido de propósito en la vida. |  |  |  |  |  |
| 1. Me siento en control de mi vida. |  |  |  |  |  |
| 1. Me gustan los desafíos. |  |  |  |  |  |
| 1. Trabajo para alcanzar mis objetivos, sin importar las dificultades en el camino. |  |  |  |  |  |
| 1. Estoy orgulloso (a) de mis logros. |  |  |  |  |  |

## **CONNOR-DAVIDSON RESILIENCE SCALE (CD-RISC)**

Tick the option you agree with to indicate your level of agreement or disagreement, among them:

- **1: Not true at all.**
- **2: A little true.**
- **3: Somewhat true.**
- **4: Mostly true.**
- **5: True all the time.**

| Items | 1 | 2 | 3 | 4 | 5 |
| --- | --- | --- | --- | --- | --- |
| 1. I am able to adapt when changes occur. |  |  |  |  |  |
| 1. I have one close and secure relationship. |  |  |  |  |  |
| 1. Sometimes fate or God helps me. |  |  |  |  |  |
| 1. I can deal with whatever comes my way. |  |  |  |  |  |
| 1. Past successes give me confidence. |  |  |  |  |  |
| 1. I try to see the humorous side of things when I am faced with problems. |  |  |  |  |  |
| 1. Having to cope with stress can make me stronger. |  |  |  |  |  |
| 1. I tend to bounce back, after illness, injury or other hardships. |  |  |  |  |  |
| 1. I believe most things happen for a reason. |  |  |  |  |  |
| 1. I make my best effort, no matter what. |  |  |  |  |  |
| 1. I believe I can make my goals, even if there are obstacles. |  |  |  |  |  |
| 1. Even when hopeless, I do not give up. |  |  |  |  |  |
| 1. In times of stress, I know where to find help. |  |  |  |  |  |
| 1. Under pressure, I stay focused and think clearly. |  |  |  |  |  |
| 1. I prefer to take the lead in problem solving. |  |  |  |  |  |
| 1. I am not easily discouraged by failure. |  |  |  |  |  |
| 1. I think of myself as a strong person when dealing with life’s challenges and difficulties. |  |  |  |  |  |
| 1. I make unpopular or difficult decisions. |  |  |  |  |  |
| 1. I am able to handle unpleasant or painful feelings like sadness, fear, and anger. |  |  |  |  |  |
| 1. I have to act on a hunch. |  |  |  |  |  |
| 1. I have a strong sense of purpose in life. |  |  |  |  |  |
| 1. I feel like I am in control. |  |  |  |  |  |
| 1. I like challenges. |  |  |  |  |  |
| 1. I work to attain goals. |  |  |  |  |  |
| 1. I take pride in my achievements. |  |  |  |  |  |

**ESCALA MULTIDIMENSIONAL DE APOYO SOCIAL PERCIBIDO**

Lea cada una de las siguientes frases cuidadosamente. Indique que tan de acuerdo está con cada una de ellas empleando esta escala:

| 1 | 2 | 3 | 4 | 5 | 6 | 7 |
| --- | --- | --- | --- | --- | --- | --- |
| Totalmente de desacuerdo | Bastante en desacuerdo | Más bien en  desacuerdo | Ni de  acuerdo ni  en  desacuerdo | Más bien  de  acuerdo | Bastante  de  acuerdo | Totalmente  de  acuerdo |

| 1. Hay una persona que está cerca cuando estoy en una situación difícil. | 1 | 2 | 3 | 4 | 5 | 6 | 7 |
| --- | --- | --- | --- | --- | --- | --- | --- |
| 2. Existe una persona especial con la cual yo puedo compartir penas y alegrías. | 1 | 2 | 3 | 4 | 5 | 6 | 7 |
| 3. Mi familia realmente intenta ayudarme. | 1 | 2 | 3 | 4 | 5 | 6 | 7 |
| 4. Obtengo de mi familia la ayuda y el apoyo emocional que necesito. | 1 | 2 | 3 | 4 | 5 | 6 | 7 |
| 5. Existe una persona que realmente es una fuente de bienestar para mí. | 1 | 2 | 3 | 4 | 5 | 6 | 7 |
| 6. Mis amigos realmente tratan de ayudarme. | 1 | 2 | 3 | 4 | 5 | 6 | 7 |
| 7. Puedo contar con mis amigos cuando las cosas van mal. | 1 | 2 | 3 | 4 | 5 | 6 | 7 |
| 8. Yo puedo hablar de mis problemas con mi familia. | 1 | 2 | 3 | 4 | 5 | 6 | 7 |
| 9. Tengo amigos con los que puedo compartir las penas y alegrías. | 1 | 2 | 3 | 4 | 5 | 6 | 7 |
| 10. Existe una persona especial en mi vida que se preocupa por mis sentimientos. | 1 | 2 | 3 | 4 | 5 | 6 | 7 |
| 11. Mi familia se muestra dispuesta a ayudarme para tomar decisiones. | 1 | 2 | 3 | 4 | 5 | 6 | 7 |
| 12. Puedo hablar de mis problemas con mis amigos. | 1 | 2 | 3 | 4 | 5 | 6 | 7 |

**MULTIDIMENSIONAL SCALE OF PERCEIVED SOCIAL SUPPORT**

Read each statement carefully. Indicate how you feel about each statement:

| 1 | 2 | 3 | 4 | 5 | 6 | 7 |
| --- | --- | --- | --- | --- | --- | --- |
| Very Strongly Disagree | Strongly Disagree | Mildly disagree | Neutral | Mildly Agree | Strongly Agree | Very Stronly Agree |

| 1. There is a special person who is around when I am in need. | 1 | 2 | 3 | 4 | 5 | 6 | 7 |
| --- | --- | --- | --- | --- | --- | --- | --- |
| 2. There is a special person with whom I can share joys and sorrows. | 1 | 2 | 3 | 4 | 5 | 6 | 7 |
| 3. My family really tries to help me. | 1 | 2 | 3 | 4 | 5 | 6 | 7 |
| 4. I get the emotional help and support I need from my family. | 1 | 2 | 3 | 4 | 5 | 6 | 7 |
| 5. I have a special person who is a real source of comfort to me. | 1 | 2 | 3 | 4 | 5 | 6 | 7 |
| 6. My friends really try to help me. | 1 | 2 | 3 | 4 | 5 | 6 | 7 |
| 7. I can count on my friends when things go wrong. | 1 | 2 | 3 | 4 | 5 | 6 | 7 |
| 8. I can talk about my problems with my family. | 1 | 2 | 3 | 4 | 5 | 6 | 7 |
| 9. I have friends with whom I can share my joys and sorrows. | 1 | 2 | 3 | 4 | 5 | 6 | 7 |
| 10. There is a special person in my life who cares about my feelings. | 1 | 2 | 3 | 4 | 5 | 6 | 7 |
| 11. My family is willing to help me make decisions. | 1 | 2 | 3 | 4 | 5 | 6 | 7 |
| 12. I can talk about my problems with my friends. | 1 | 2 | 3 | 4 | 5 | 6 | 7 |
